# Change in crown inclination accompanying initial tooth alignment with round archwires

**DOI:** 10.1590/2177-6709.27.3.e2220489.oar

**Published:** 2022-07-04

**Authors:** Mona A. MONTASSER, Ludger KEILIG, Christoph BOURAUEL

**Affiliations:** 1 Mansoura University, Faculty of Dentistry, Orthodontic Department (Mansoura/Egypt).; 2 University of Bonn, Department of Oral Technology, School of Dentistry (Bonn/Germany).

**Keywords:** Inclination, Tooth alignment, Orthodontic archwires, Orthodontic brackets

## Abstract

**Objective:**

To evaluate, in-vitro, the change in crown inclination that occurs during orthodontic leveling and alignment using different archwire-bracket-ligation combinations.

**Materials and Methods:**

Four archwire types were tested: (1) 0.012-in stainless steel and (2) 0.0155-in stainless steel multi-stranded, (3) 0.012-in nitinol Orthonol® and (4) 0.012-in nitinol Thermalloy®. Combinations with five types of 0.022-in slot orthodontic brackets were tested: SmartClip^TM^ and Time3® self-ligating brackets, Mini-Taurus® and Victory Series^TM^ conventional brackets, and Synergy® conventional-low friction bracket. Conventional brackets were ligated with both stainless steel and elastomeric ligatures. The simulated malocclusion comprised 2.0mm gingival and 2.0mm labial displacements of a maxillary right central incisor. Rotation around the Y-axis (representing labio-palatal inclination) was measured for the different archwire-bracket-ligation combinations.

**Results:**

The largest rotation was measured whith Orthonol® and Thermalloy® wires when combined with SmartClip^TM^ brackets (8.07±0.24º and 8.06±0.26º, respectively) and with Synergy® brackets ligated with stainless steel ligatures (8.03±0.49º and 8.0±0.37º, respectively). The lower rotation was recorded when Thermalloy®, multi-stranded, and Orthonol® wires were ligated with elastomeric rings to Mini-Taurus® brackets (1.53±0.18º, 1.65± 0.23º and 1.70±0.28º, respectively) and to Victory Series^TM^ brackets (1.68± 0.78º, 2.92± 1.40º and 1.74±0.46º, respectively).

**Conclusions:**

All archwire-bracket-ligation combinations produced lingual crown inclination; however, lower changes were observed when the conventional brackets were ligated with elastomeric rings. The multi-stranded archwire produced less rotation with nearly every bracket-ligation combination, compared to the other archwires. The effect of the archwire-bracket-ligation combination on tooth inclination during leveling and alignment should be considered during planning treatment mechanics.

## RESUMO

**Objetivo:**

Avaliar, *in vitro*, as mudanças na inclinação das coroas dentárias que acontecem durante o alinhamento e nivelamento usando diferentes combinações de arcos/braquetes/ligaduras.

**Material e Métodos:**

Foram usados quatro tipos de arcos ortodônticos: (1) 0,012” de aço inoxidável, (2) 0,0155” de aço multifilamentado, (3) Orthonol® 0,012” de nitinol e (4) Thermalloy® 0,012” de nitinol. Esses arcos foram combinados com cinco tipos de braquetes ortodônticos com *slot* 0,022”: braquetes autoligáveis SmartClip^TM^ e Time3®, braquetes convencionais Mini-Taurus® e Victory Series^TM^, e braquetes convencionais de baixo atrito Synergy®. Os braquetes convencionais receberam ligaduras de aço ou elastoméricas. A má oclusão simulada foi composta por um deslocamento do incisivo central superior direito em 2 mm para a cervical e 2 mm para a vestibular. A rotação ao redor do eixo Y (representando a inclinação vestibulolingual) foi medida para as diferentes combinações de arco/braquete/ligadura.

**Resultados:**

A maior rotação foi medida quando os arcos Orthonol® e Thermalloy® foram usados com os braquetes SmartClip^TM^ (8,07±0,24º e 8,06±0,26º, respectivamente) e com os braquetes Synergy® com ligaduras metálicas (8,03±0,49º e 8,0±0,37º, respectivamente). A menor rotação foi vista quando os arcos Thermalloy®, multifilamentado e Orthonol® foram presos por ligaduras elastoméricas aos braquetes Mini-Taurus® (1,53±0,18º, 1,65±0,23º e 1,70±0,28º, respectivamente) e aos braquetes Victory Series^TM^ (1,68±0,78º, 2,92±1,40º e 1,74±0,46º, respectivamente).

**Conclusões:**

todas as combinações arco-braquete-ligadura produziram inclinação palatina da coroa. Entretanto, as menores mudanças foram observadas quando os braquetes convencionais receberam ligaduras elastoméricas. O arco multifilamentado produziu menor rotação com quase todas as combinações braquete-ligadura, em comparação aos outros arcos. O efeito da combinação arco-braquete-ligadura na inclinação dentária durante o alinhamento e nivelamento deve ser considerado durante o planejamento da mecânica dos tratamentos.

## INTRODUCTION

Andrews,^1^ in his prominent article introducing the six keys to normal occlusion, described the importance of adjusting the maxillary incisors’ labio-palatal inclination to achieve normal overbite and proper posterior occlusion. Since then, controlling maxillary incisors’ inclination for the achievement of pleasing esthetics and convenient occlusion has been well established in Orthodontics.^2^ In-vitro and in-vivo studies have investigated the relationship between tooth inclination and arch perimeter^3^, smile and facial features,^4^ ^,^ ^5^ forces and moments generated,^6^ ^,^ ^7^, and perceived tooth color.^8^ Emphasis has shifted in Orthodontics from molars to incisors; at first to the mandibular incisors and then to the maxillary incisors. This shift happened with advances in orthodontic and orthognathic techniques that made the position of the maxillary incisors the start point in certain treatment plans.^9^

The crown inclination is a feature that could be manipulated with rectangular archwires at the finishing stage of orthodontic treatment by creating a moment generated by the torsion of a rectangular archwire in the bracket slot.^10^ This action requires full-size archwires to avoid play between the archwire and the slot walls.^11^ ^,^ ^12^ However, archwire of a round cross-section is the standard choice for initial leveling and alignment.^13^ Round archwires produce tipping movement, thus changing crown inclination, which may be or may not be desired. Archwire material choices for leveling and alignment include, but are not limited to, stainless steel and nitinol.^14^

Thus, the objective of this study was to evaluate, in-vitro, the change in crown inclination that occurs during the orthodontic leveling and alignment of a displaced central incisor using different archwire-bracket-ligation combinations.

## MATERIAL AND METHODS

Material: Four archwire types; two stainless steel (1) 0.012-in stainless steel (3M Unitek, Monrovia, Ca) and (2) 0.0155-in multi-stranded (Advanced Orthodontics, Näpflein GmbH, Düsseldorf, Germany); and two nitinol wires (3) 0.012-in Orthonol^®^ (Rocky Mountain Orthodontics, Denver, Co) and (4) 0.012-in Thermalloy^®^ (Rocky Mountain Orthodontics, Denver, Co) were used in combination with five types of 0.022-in slot orthodontic brackets: (1) SmartClip^TM^ (3M Unitek, Monrovia, Ca) passive self-ligating bracket, (2) Time3^®^ (American Orthodontics, Sheboygan, WI) active self-ligating bracket, (3) Mini-Taurus^®^ (Rocky Mountain Orthodontics, Denver, Co) conventional bracket, (4) Victory Series^TM^ (3M Unitek, Monrovia, Ca) conventional bracket, and (5) Synergy^®^ (Rocky Mountain Orthodontics, Denver, Co) conventional low-friction bracket. Two ligation methods were used: (1) Stainless steel wire ligatures and (2) Elastomeric rings. Each archwire-bracket-ligation combination was tested 20 times using a new archwire each time.

Testing machine: The Orthodontic Measurement and Simulation System (OMSS) is based on two sensors capable of 3D-recording of forces and moments. The OMSS is built in a temperature-controlled compartment. This is especially important for heat-managed alloys; therefore, during testing the nitinol archwires the temperature was kept at 37±1°C. Further details about the OMSS and its applications in orthodontics can be found in articles devoted to that purpose.^15^ ^,^ ^16^

Preparing the measurement setup: Brackets were bonded with a cyanoacrylate adhesive from the right second premolar to the left second premolar of fabricated maxillary arch resin models. These resin models had the right central incisor removed to create a space for a bracket holder that would be connected to one of the sensors of the OMSS. The tested archwire was then tied to the conventional brackets with either stainless steel ligatures or elastomeric rings. The ligature wires or the elastomeric rings were tied around the two central wings of the six wings of the Synergy^®^ bracket. Tying the steel ligatures around the bracket wings was short one turn from complete tightening, to ensure free play between the wire and the brackets. In the case of self-ligating brackets, the archwire was inserted and then the clips or doors of the self-ligating brackets were closed. The right central incisor bracket was then bonded to a bracket holder using a jig to standardize the position to be repeated with every bracket. The design of the setup included positioning the center of resistance of the simulated misaligned central incisor at 10.0mm apically and 4.5mm lingual to the point of the force application. This position was set based on the design of the whole setup and the position of the bracket and the bracket holder, representing the simulated displaced central incisor.

**Figure 1:**
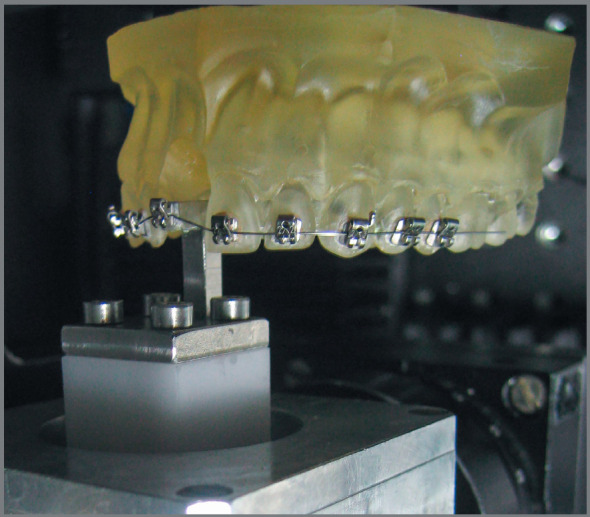
Representation of the intended simulation; 2.0mm labial and 2.0mm gingival displacements.

Measurement procedures: Resin model was fixed in its position of the OMSS, and the bracket holder, with the right central incisor bracket bonded to it, was fixed to one of the force sensors of the OMSS (Fig 1). The sensor was adjusted to occupy the place of the removed right central incisor, aligning the bracket with the rest of the brackets bonded to the resin model. At this point, the archwire was tied to the bracket on the holder. The whole assembly now simulated an aligned arch. With this arrangement, the labio-palatal rotation would be along the Y-axis. The OMSS was set to move the bracket holder from the initial position 2.0mm in the gingival (X-axis) and 2.0mm in labial (Z-axis) directions, and then move in reverse to the initial position in 0.01mm increments. The bracket holder was adjusted so the initial forces and moments recorded by the sensor were as close to zero as possible. This start position was reproduced for each measurement.

Data extraction: Data was transferred from the OMSS software to Excel® sheets. 

Data organization and statistical analyses: The maximum absolute values of rotation around the Y-axis were arranged in the Excel^®^ sheets, and descriptive statistics including means and standard deviations were calculated for each archwire-bracket-ligation combination. Raw data was tested for normality and, as the data revealed normal distribution, a two-way analysis of variance ANOVA followed by Tukey Post-Hoc Test and Student *t*-test was employed using IBM SPSS Statistics for Windows (version 20.0, IBM Corp., Armonk, NY).

## RESULTS

The results of two-way ANOVA (Table 1) revealed a statistically significant effect of the bracket (F = 132.759, *p*< 0.001) and the archwire (F = 28.836, *p*< 0.001) selections on the rotation of the tooth around the Y-axis, and also a significant interaction between bracket type and archwire type (F = 8.609, *p*< 0.001).

**Table 1:** Results of two-way ANOVA.

Two-way ANOVA				
	Sum of squares	df	F	P value
Brackets	324.405	7	132.759	< 0.001*
Archwires	70.463	3	28.836	< 0.001*
Archwires × Brackets	21.036	21	8.609	< 0.001*

* Statistically significant for *p* ≤ 0.05.

From the pairwise comparisons (Table 2) between different brackets, when used with the same archwire and different ligation methods, noticeable less rotation was observed with conventional brackets ligated with elastomeric rings, compared to all other combinations. Comparing rotation produced by one archwire type ligated with steel ligatures *versus* elastomeric rings to a conventional bracket, showed a significant difference in the case of Mini-Taurus^®^ and Victory Series^TM^ brackets but, not in the case of Synergy^®^ brackets. Pairwise comparisons (Table 3) revealed that different archwires produced different effects when combined with the same bracket type ligated in the same way, except when archwires were combined with Synergy^®^ brackets ligated with elastomeric rings (*p*=0.466) and with Time3^®^ (*p*=0.465). 

**Table 2:** Pairwise comparisons between rotations produced by the same leveling archwire when combined with the different brackets.

Wire type	Bracket Type								F	P
	Conventional brackets						Self-ligating brackets			
	Steel-ligation			Elastic-ligation						
	Mini-Taurus^®^ (n = 20)	Victory Series^TM^ (n = 20)	Synergy^®^ (n = 20)	Mini-Taurus^®^ (n = 20)	Victory Series^TM^ (n = 20)	Synergy^®^ (n = 20)	SmartClip^TM^ (n = 20)	Time3^®^ (n = 20)		
Stainless steel	-7.57 ± 0.59^c^	-6.99 ± 0.44^c^	-7.53 ± 0.91^c^	-5.98 ± 0.55^b^	-4.91 ± 0.67^a^	-7.29 ± 1.49^c^	-7.09 ± 1.22^c^	-7.80 ± 0.96^c^	22.501*	<0.001*
Multi- stranded	-5.33 ± 3.27^c^	-5.92 ± 1.88^c^	-5.1 ± 0.48^b,c^	-1.80 ± 0.58^a^	-2.9 ± 1.40^ab^	-7.27 ± 2.01^c^	-6.59 ± 1.32^c^	-7.36 ± 4.79^c^	14.147*	<0.001*
Orthonol^®^	-7.36 ± 0.43^b,c,d^	-7.24 ± 0.9^b,c^	-7.93 ± 1.1^cd^	-2.06 ± 0.81^a^	-1.74 ± 0.46^a^	-7.9 ± 1.01^cd^	-8.07 ± 0.31^d^	-6.63 ± 1.25^b^	192.45*	<0.001*
Thermalloy^®^	-7.14 ± 3.52^d^	-6.66 ± 1.40^b^	-7.83 ± 1.31^b^	-1.47 ± 0.30^a^	-1.68 ± 0.78^a^	-7.69 ± 1.13^b^	-8.07 ± 0.36^b^	-6.78 ± 1.42^b^	58.933*	<0.001*

F: F for ANOVA test, pairwise comparison between each two groups was done using Tukey’s *post-hoc* test.

*p*: *p*-value for comparison between the different brackets with the same archwire.

* Statistically significant at p ≤ 0.05.

Means with the same letters are not significant (i.e. means with different letters are significant).

**Figure 2:**
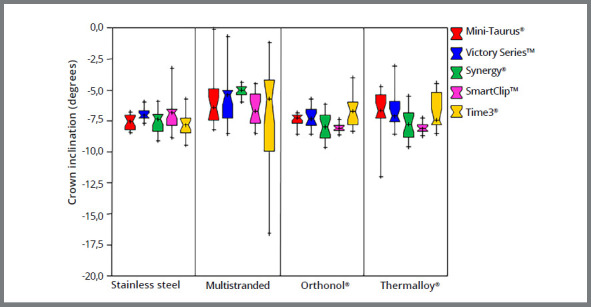
Change in central incisor crown inclination by different archwires ligated to self-ligating and conventional brackets with stainless steel ligatures.

**Figure 3:**
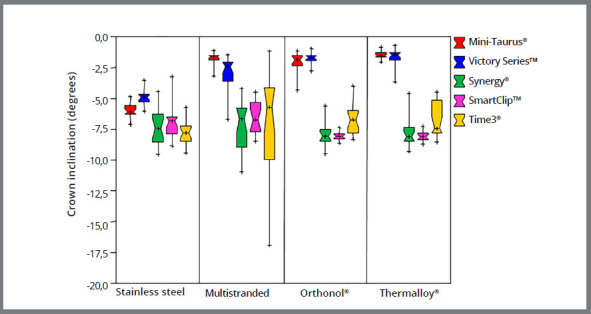
Change in central incisor crown inclination by different archwires ligated to self-ligating and conventional brackets with elastic ligatures.

The boxplots compare changes in crown inclination produced by self-ligating brackets to conventional brackets ligated with stainless steel ligatures (Fig 2) and elastomeric rings (Fig 3). The figures show that, contrary to the two other conventional brackets, the low-friction Synergy^®^ bracket ligated with elastomeric rings produced rotation comparable to that produced by self-ligating brackets and conventional brackets ligated with stainless steel ligatures. 

## DISCUSSION

The registered changes in the maxillary incisor inclination in the present study varied significantly between the different archwire-bracket-ligation combinations; however, the direction of the change was constantly negative (-). According to the sign system proposed by Burstone and Koenig^17^, the forces acting in the mesial, labial or buccal, and incisal or occlusal directions are positive (+), while forces acting in distal, lingual or palatal, and gingival directions are negative (-). Similarly, moments (couples) producing mesial and labial or buccal crown movements are positive (+), and moments producing distal, and lingual or palatal crown movements are negative (-). Applying these rules on the experimental setup of the present study, the positive (+) rotation would be a labial crown inclination, while the negative (-) rotation would be lingual crown inclination. The simulated malocclusion of 2.0mm gingival and 2.0mm labial displacements resulted in incisal and palatal forces at the bracket. The palatal force generates a negative moment, tending to rotate the crown palatally, and the extruding force generates a negative moment, rotating the crown palatally. The net result is a negative moment resulting from the forces and lever arms. The simulated malocclusion in the current study was corrected by round wires that cannot create a counteracting moment.

Evaluating changes in crown inclination produced by each bracket when combined with the different archwires, as seen in Table 2, less rotation was produced by conventional brackets ligated with elastomeric rings. Compared to the two other conventional brackets, Synergy^®^ brackets ligated with elastomeric rings produced comparable or more rotation. In the current study, the elastomeric ring was tied around the two central wings of the Synergy^®^ bracket, which are raised, preventing the contact between the archwire and the ligature and thus ensuring free play between the archwire and the bracket slot walls. Meanwhile, steel ligature was tied slightly loose because this is the preferred method during leveling and alignment to allow free sliding and efficient tooth movement with less force exerted on the tooth. The play between the wire and the bracket slot is probably the most important factor influencing the labio-palatal inclination of teeth.^18^ Multi-stranded archwires produced less rotation with every bracket-ligation combination. The rotation was significantly higher when Mini-Taurus^®^ and Victory Series^TM^ brackets were ligated with stainless steel ligatures than elastomeric rings, but Synergy^®^ brackets showed insignificant difference and significantly higher rotation with multi-stranded archwire ligated with elastomeric rings. Because of the nature of this in-vitro study, new elastomeric rings were used for ligating each archwire. Controlling rotation could be the result of the seating force of the new elastomeric on the archwire. However, elastomeric rings deteriorate quickly in the oral cavity to the extent that they have been found to lose 40% of their force in the first 24 hours.^19^ Frequent change of the elastomeric rings might be needed to overcome material deterioration and keep the control on rotation. Tight steel ligature would be required.

There was no statistical difference, as shown in Table 3, in rotation produced by any of the archwires used in the current study between SmartClip^TM^ a passive self-ligating bracket, and Synergy^®^ a low friction conventional bracket whether ligated with stainless steel or elastomeric rings. The design of the Synergy^®^ bracket and the method of ligating the archwire around the central wings (elastomeric rings or loose steel ligatures) made it comparable to the passive self-ligating bracket. The rotation produced by the two types of steel archwires was higher with Time3^®^ brackets than SmartClip^TM^ brackets, but differences were not significant. The rotation that was produced by the two types of nitinol archwires was higher with SmartClip^TM^ brackets than Time3^®^ brackets, and the difference was significant in case of Orthonol^®^ archwire. It is noted that the active self-ligating Time3^®^ brackets showed a wide range of rotation values with multi-stranded and Thermalloy^®^ archwires, this evidence could be attributed to the interaction of the clip with these archwire materials while exerting a seating force on the archwire.

**Table 3:** Pairwise comparisons between rotations produced by the different leveling archwires when combined with the same bracket type

Ligation	Brackets	Archwires			F	p	
		Stainless steel (n = 20)	Multi-stranded (n = 20)	Orthonol® (n = 20)	Thermalloy^®^ (n = 20)		
Steel-ligation	Mini-Taurus^®^	-7.57 ± 0.59^b^	-5.33 ± 3.27^a^	-7.36 ± 0.43^b^	-7.14 ± 3.52^ab^	3.578	0.018*
	Victory Series^TM^	-6.99 ± 0.44^b^	-5.92 ± 1.88^a^	-7.24 ± 0.90^b^	-6.66 ± 1.40^ab^	4.014	0.010*
	Synergy^®^	-7.53 ± 0.91^b^	-5.08 ± 0.48^a^	-7.93 ± 1.13^b^	-7.83 ± 1.31^b^	36.201	<0.001*
Elastic-ligation	Mini-Taurus^®^	-5.98 ± 0.55^c^	-1.80 ± 0.58^ab^	-2.06 ± 0.81^b^	-1.47 ± 0.30^a^	258.88	<0.001*
	Victory^TM^	-4.91 ± 0.67^c^	-2.92 ± 1.40^b^	-1.74 ± 0.46^a^	-1.68 ± 0.78^a^	56.403	<0.001*
	Synergy^®^	-7.29 ± 1.49^a^	-7.27 ± 2.01^a^	-7.88 ± 1.01^a^	-7.69 ± 1.13^a^	0.860	0.466
Self-ligation	SmartClip^TM^	-7.09 ± 1.22^b^	-6.59 ± 1.32^b^	-8.07 ± 0.31^a^	-8.07 ± 0.36^a^	12.588	<0.001*
	Time3^®^	-7.80 ± 0.96^a^	-7.36 ± 4.79^a^	-6.63 ± 1.25^a^	-6.78 ± 1.42^a^	0.861	0.465

F: F for ANOVA test, pairwise comparison between each two groups was done using Tukey’s *post-hoc* test.

*p*: *p*-value for comparison between the different leveling archwires with the same bracket.

* Statistically significant at p ≤ 0.05.

Means with the same letters are not significant (i.e. means with different letters are significant).

The change in crown inclination in the present study is the result of a rotational moment dependent on the force applied on the tooth and the distance from that point of force application to the designated center of resistance of the simulated misaligned central incisor. In this study design, the distance was standardized for all tests, therefore the changing factor is the force applied to the tooth from each archwire-bracket-ligation combination. These archwire-bracket-ligation combinations showed a significant interaction between the archwire and the bracket type, and a consistently higher force applied on the displaced central incisor when elastomeric rings were used, which consequently affected the correction of the malocclusion.^20^ ^,^ ^21^

Orthodontic tooth movement is the result of a combination of many mechanical factors related to the bracket-archwire-ligation combination, including: bracket prescription, bracket slot dimension, bracket material, archwire dimension, archwire material, inter-bracket distance, and method of ligation (stainless steel wire, elastomeric rings or self-ligation).^22^ ^-^ ^26^ The oral environment, however, adds additional elements to these factors. Saliva, thermocycling, and forces of occlusion lead to aging and deterioration of the orthodontic materials. Anisotropic periodontal ligament may also complicate the prediction of tooth movement.^27^ Even with the application of the same force system, different responses are expected depending on the surrounding environment and involved biological factors that make tooth movement occurs in a certain plane more easily than it occurs in the other planes.^28^ Since mechanics is a major factor that affects force/moment systems from the start of treatment, controlling tooth inclination should be planned from the leveling and alignment because of the different requirements among the types of malocclusion and the individual cases in each malocclusion type. Manipulating tooth inclination from the start would help clinicians to limit the need for bends and other auxiliaries in later stages. 

Clinically, multiple teeth or arch segments involved in the malocclusion will lead to complex force systems that sometimes might be hard to predict. Realizing the true complexity of the force systems that could result from the used biomechanics in the oral cavity would help clinicians to use biomechanics more efficiently and simply.^28^

Clinical relevance: Results could be useful as a guide when comparing orthodontic materials and planning biomechanics. Results contribute primarily to the understanding of the performance of an orthodontic appliance and how the outcomes are affected by each of the appliance components, as well as the interplay among the components that are in the present study: archwire, bracket, and ligation.

## CONCLUSIONS

1. Conventional brackets ligated with elastomeric rings produced the smallest rotation; however, low-friction conventional brackets did not show similar behavior.
2. Passive self-ligating bracket and low-friction conventional bracket produced comparable rotation.
3. Multi-stranded archwires produced less rotation with every bracket-ligation combination, compared to the other archwires.
4. Controlling tooth inclination could start from the leveling and alignment stage by careful selection of the archwire-bracket-ligation combination.
